# Imaging of mandibular fractures: a pictorial review

**DOI:** 10.1186/s13244-020-0837-0

**Published:** 2020-02-19

**Authors:** Cosimo Nardi, Chiara Vignoli, Michele Pietragalla, Paolina Tonelli, Linda Calistri, Lorenzo Franchi, Lorenzo Preda, Stefano Colagrande

**Affiliations:** 1 Department of Experimental and Clinical Biomedical Sciences, Radiodiagnostic Unit n. 2, University of Florence—Azienda Ospedaliero-Universitaria Careggi, Largo Brambilla 3, 50134 Florence, Italy; 2 Department of Surgery and Translational Medicine, University of Florence, Via del Ponte di Mezzo, 46-48, 50127 Florence, Italy; 3 Department of Orthodontics and Pediatric Dentistry, School of Dentistry, University of Michigan, Ann Arbor, USA; 4 Department of Clinical-Surgical, Diagnostic and Pediatric Sciences, University of Pavia, Via Alessandro Brambilla, 74, 27100 Pavia, Italy; 5 Diagnostic Imaging Unit, National Centre of Oncological Hadrontherapy (CNAO), Pavia, Italy

**Keywords:** Mandible, Condyle, Fracture, Trauma, Panoramic radiography

## Abstract

Mandibular fractures are among the most common maxillofacial fractures observed in emergency rooms and are mainly caused by road accidents. The clinical features of mandibular fractures include malocclusion and loss of mandibular function. Panoramic radiography is usually limited to isolated lesions, whereas computed tomography is the tool of choice for all other facial traumatic events. No reference standard classification system for the different types of mandibular fractures is defined. Therapeutic options include a conservative approach or surgical treatment based on the anatomic area and the severity of fracture. The main purpose of this pictorial review is to illustrate a practical description of the pathophysiology of mandibular fractures and describe both the imaging techniques to recognise them and the therapeutic indications.

## Key points

- Mandibular fractures represent two thirds of all maxillofacial fractures.
- X-ray films, including panoramic radiography, are usually limited to mild traumatic events.
- Computed tomography is the tool of choice for the assessment of mandibular fractures.
- Knowledge of the action of masticatory muscles is crucial for recognising bone fragment displacements.
- The treatment varies depending on the anatomic area and type of fracture.

## Introduction

**Fig. 1:**
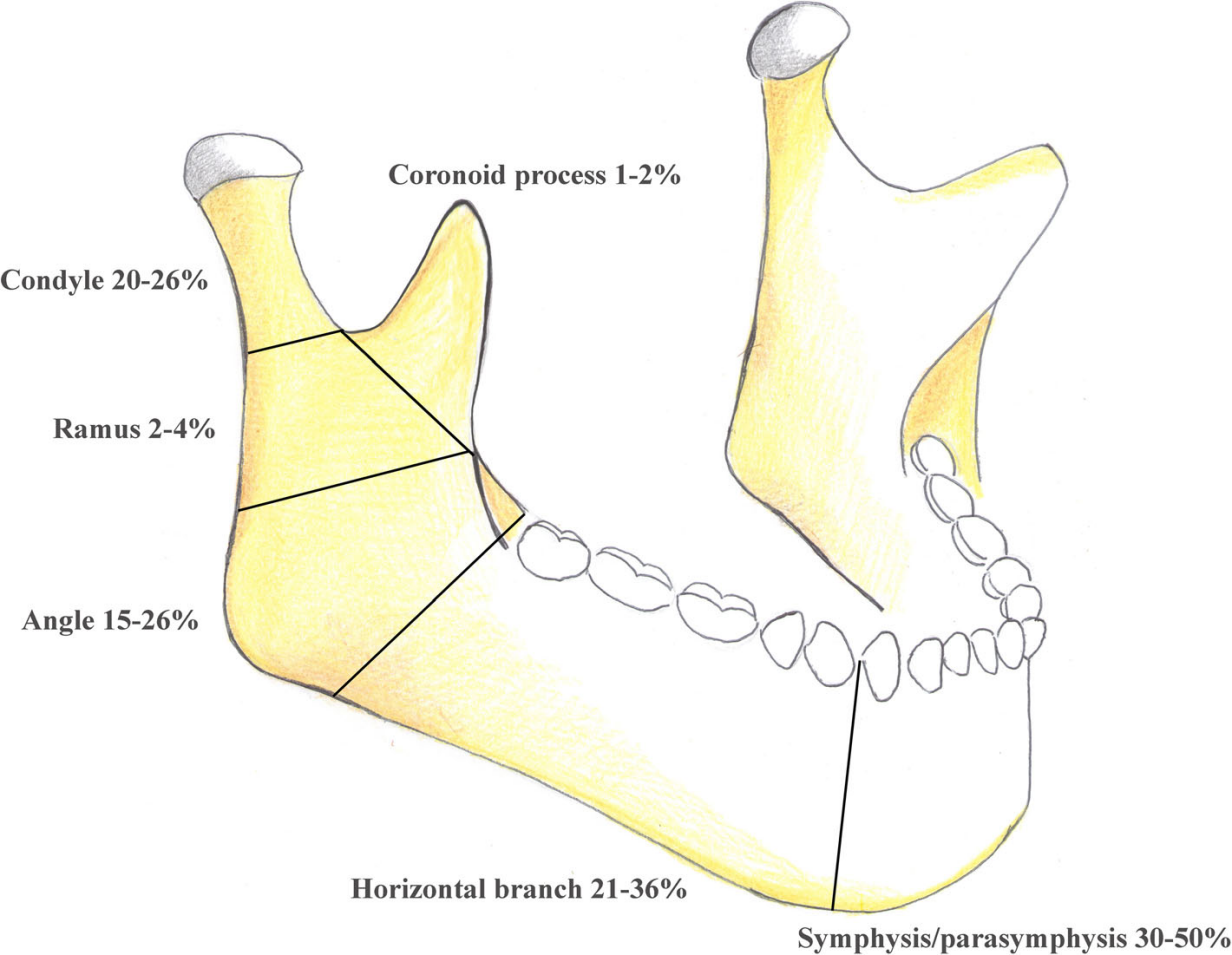
Anatomic areas of mandibular fractures

Mandibular fractures are among the most common (60–70%) maxillofacial fractures observed in emergency rooms [1]; more than 2,500 people suffer a mandibular fracture every year in the USA [2]. The epidemiology of maxillofacial fractures varies according to geographical areas and socio-economic factors. The most common causes of maxillofacial fractures are road traffic accidents (40–42%), falls, assaults, sports, and work injuries [3]. The average age of patients with mandibular fracture is 38 years for men and 40 years for women [4]. Men are mainly involved (male-to-female ratio 5:1) [5]. Mandibular fractures can be classified in relation to their anatomic localisation (Fig. 1) as follows: symphysis/parasymphysis (30–50%), horizontal branch (21–36%), angle (15–26%), ramus (2–4%), condyle (20–26%), and coronoid process (1–2%).

Mandibular fractures are found in 44.2% of patients who are admitted to emergency rooms for facial trauma [6], and only in 7% of cases is a mandibular fracture not confirmed by the findings of imaging investigations when it is clinically suspected [7].

The current pictorial essay is the first review to analyse the relevant anatomy and biomechanics of the mandible concerning the types of fracture. In addition, an overview of conservative and surgical management is reviewed.

## Imaging techniques

Radiography represents the first level imaging technique in patients with traumatic injury of the mandible. Three different X-ray views can be performed for mandibular fractures: a postero-anterior view, generally used for angle and ramus fractures; an angled antero-posterior view called reverse Towne view, useful in case of displacement of condylar fragments; bilateral oblique view, used to analyse the angle and horizontal branch of the mandible. Panoramic radiography (PAN) is a zonography of upper and lower jaws. It has much higher sensitivity than the three aforementioned X-ray view series for the detection of mandibular fractures (70–92% and 66%, respectively) [1, 8]. Unfortunately, both PAN and X-ray views are affected by the typical disadvantages of two-dimensional imaging [9]—difficulty in the patient’s positioning, anatomic noise, superimposition, geometric distortion, X-ray angulations, and radiographic contrast—and may be burdened by the slight movements of the mandible, resulting in artefacts. This is the reason why two-dimensional imaging of mandibular fractures is usually limited to isolated lesions. Multislice spiral computed tomography (MSCT) represents the reference survey in complex fractures because it benefits from thin-layer thicknesses (0.5–1.0 mm), native images, and three-dimensional multiplanar reformat reconstructions with no overlap between the different anatomic structures. MSCT has sensitivity around 100% in the detection of mandibular fractures [1].

Recently, a new three-dimensional imaging technique called cone beam computed tomography (CBCT) has proved to supply an excellent volumetric study of maxillofacial bone structures [10] and satisfactorily recognise mandibular fractures [11]. Moreover, it has a high spatial resolution (0.075–0.4 mm isotropic voxel) [12], delivers relatively low radiation doses compared to MSCT [13], and is only slightly affected by metal artefacts, which often occur in patients stabilised by immobilisation techniques that use metallic materials during post-treatment follow-up [14]. Long scan times (5.4–40 s) [15] advise against the use of CBCT in patients who experience pain and have functional disability for the increased risk of motion artefacts [16]. Nevertheless, MSCT must inevitably be recommended in multiple traumatised patients since it has a short scan time, allows better image quality for the soft tissue visualisation, and can be used for contrast-enhanced examinations [17].

Magnetic resonance imaging (MRI) is considered to be the best technique for soft tissue evaluation in condylar fractures since it can accurately identify any post-traumatic alteration of the structures that make up the temporomandibular joint, especially the displacement of mandibular condyles [18]. Furthermore, MRI is ideal for determining the increase in the amount of extracellular water in bone marrow oedema, whereas MSCT allows high-quality study of the cortical bone [19].

## Mandibular anatomy, function, and fractures

### Mandibular body

**Fig. 2:**
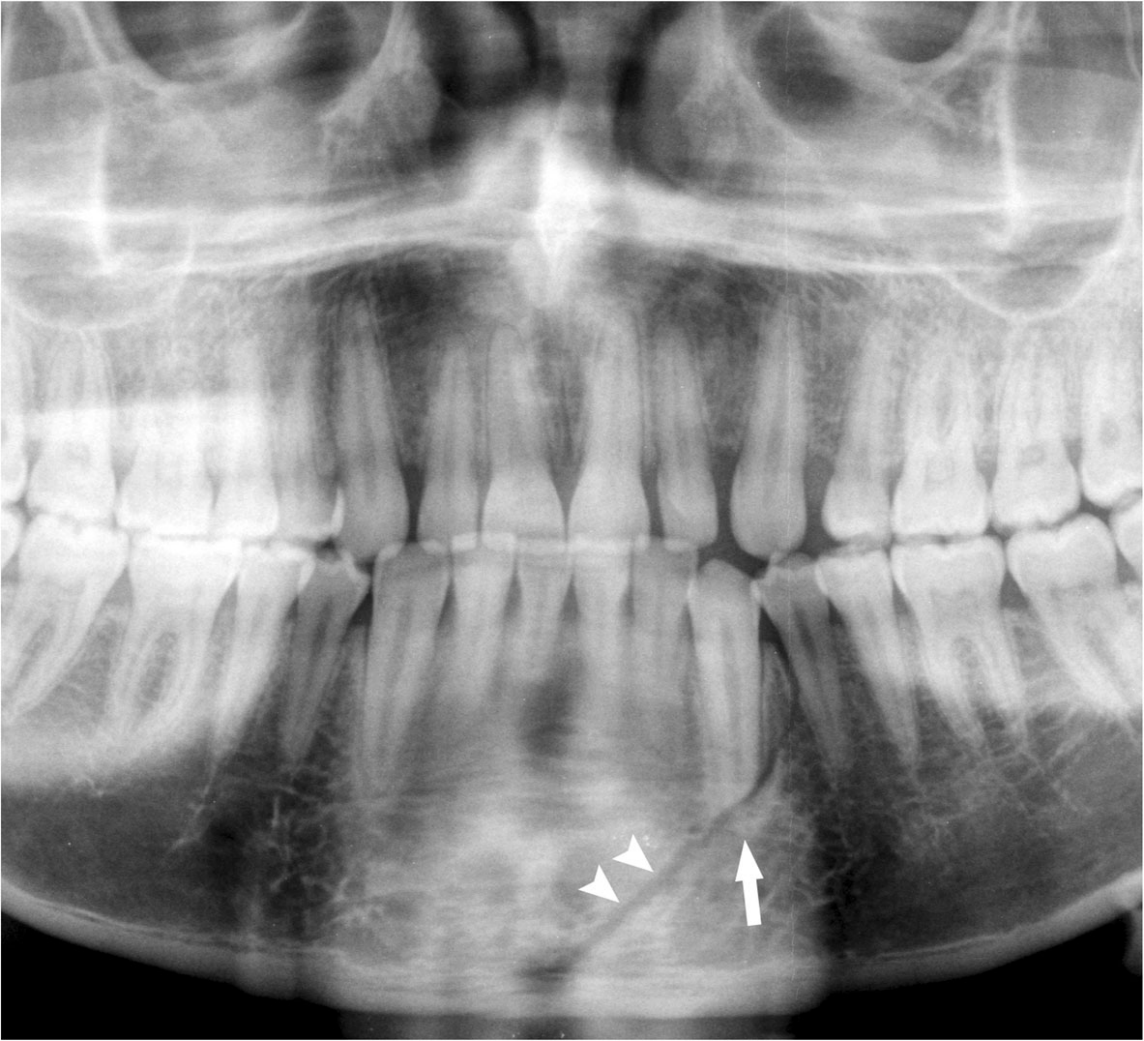
Symphysis fracture. Cropped panoramic radiograph. The fracture rhyme (arrowheads) runs from the base of the mandibular symphysis to the alveolar process of the lower left first premolar. The root apex of the canine is fractured (arrow)

**Fig. 3:**
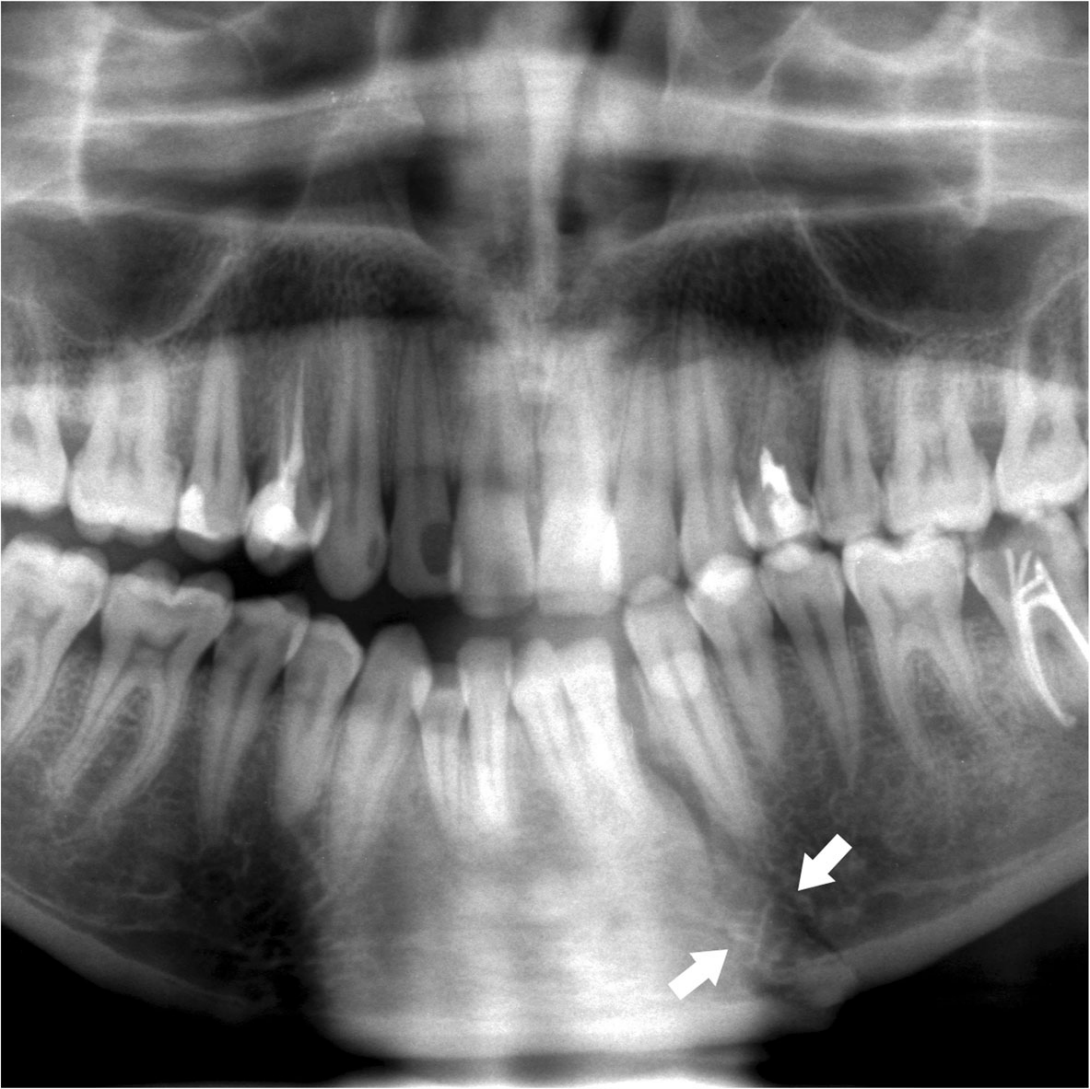
Symphysis fracture with lambda course. Cropped panoramic radiograph. Two rhymes of fracture (arrows) converge in the area between the lower left lateral incisor and the canine

The mandibular body is shaped like a horseshoe, with a concave internal face. From the lower lingual part of the symphysis originate the mylohyoid, geniohyoid, and anterior belly of digastric muscles that are inserted on the hyoid bone. The upper edge of the mandibular body has sixteen alveolar cavities, varying in size and depth according to the tooth roots. Impacted teeth or teeth with long roots, such as canines, generate lines of weakness and make mandibular fractures easier [20]. Fractures of the mandibular body include fractures of the symphysis/parasymphysis and horizontal branches. The symphysis/parasymphysis area corresponds to the region between the two canines. To simplify our analysis, the generic term symphysis refers to both the symphysis and parasymphysis areas [21]. The symphysis fracture rhyme can be median or paramedian (Fig. 2) and can have a rectilinear or lambda course (Fig. 3).

**Fig. 4:**
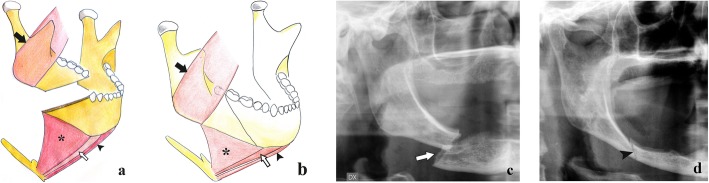
Horizontal branch fractures. **a** Unfavourable fracture. Picture showing a fracture with a downward and posterior direction. The bone fragments are misaligned by the action of the masseter muscle (black arrow) that pulls the distal bone fragment upwards, and the mylohyoid (asterisk), geniohyoid (white arrow), and anterior belly of digastric (black arrowhead) muscles that pull the mesial bone fragment downwards. **b** Favourable fracture. Picture showing a fracture with a downward and anterior direction. The bone fragments impact each other with no displacement. **c**, **d** Cropped panoramic radiographs in toothless patients with unfavourable (arrow) and favourable (arrowhead) horizontal branch fractures

Fractures of the horizontal branch are located in the area between the canine and mandibular angle. These fractures can be qualified as unfavourable or favourable on the basis of the direction of the fracture rhyme and the muscle attachment points that lead to displacement or no displacement of bone fragments, respectively (Fig. 4). The masseter, temporal, and medial pterygoid muscles pull the horizontal branch upwards, whereas the digastric, geniohyoid, and mylohyoid muscles move the mandibular symphysis downwards. Therefore, the fracture is unfavourable when the fracture rhyme runs from the alveolar ridge to the lower mandibular cortex with a posterior direction since the bone fragments are displaced. On the contrary, the fracture is favourable when the fracture rhyme runs anteriorly since the bone fragments are moved towards each other with no displacement [22].

### Mandibular angle

The mandibular angle is defined as the angle formed by the junction of the lower edge of the ramus and the external face of the mandibular body. Mandibular angle fractures occur in a triangular area included between the anterior edge and the postero-superior insertion of the masseter muscle. These fractures are distal to the third molar and are often found in cases of personal aggression [23]. The masseter and medial pterygoid muscles elevate the mandible and are inserted at the external and internal faces of the mandibular angle respectively (Table 1).

**Table 1:** Insertion points and actions of the masticatory muscles

Muscle	Proximal attachment	Distal attachment	Action on the mandible	Action on bone fragments
Lateral pterygoid	Greater wing of sphenoid (upper head) and lateral pterygoid plate (lower head).	Pterygoid fovea, temporomandibular joint, and articular disc.	Protrudes the mandible. The synergistic action of the bilateral lateral pterygoid muscles contributes to the opening of the mandible.	In condylar neck fractures, it pulls the condylar head anteriorly and medially.
Medial pterygoid	Lateral pterygoid plate, pyramidal process of palatine bone, and maxillary tuberosity.	Ramus and angle of the mandible.	Protrudes and elevate the mandible. The medial pterygoid, temporal, and masseter muscles close the mandible.	In mandibular angle fractures, it elevates the proximal bone fragment.
Temporal	Temporal fossa and temporal fascia.	Coronoid process and ramus of the mandible.	Retrudes and elevates the mandible. The medial pterygoid, temporal, and masseter muscles close the mandible.	In coronoid process fractures, it elevates and retracts the apical bone fragment.
Masseter	Zygomatic arch.	Coronoid process and ramus of the mandible.	Protrudes, retrudes, and elevates the mandible. The medial pterygoid, temporal, and masseter muscles close the mandible.	In horizontal branch or mandibular angle fractures, it elevates the distal bone fragment.

**Fig. 5:**
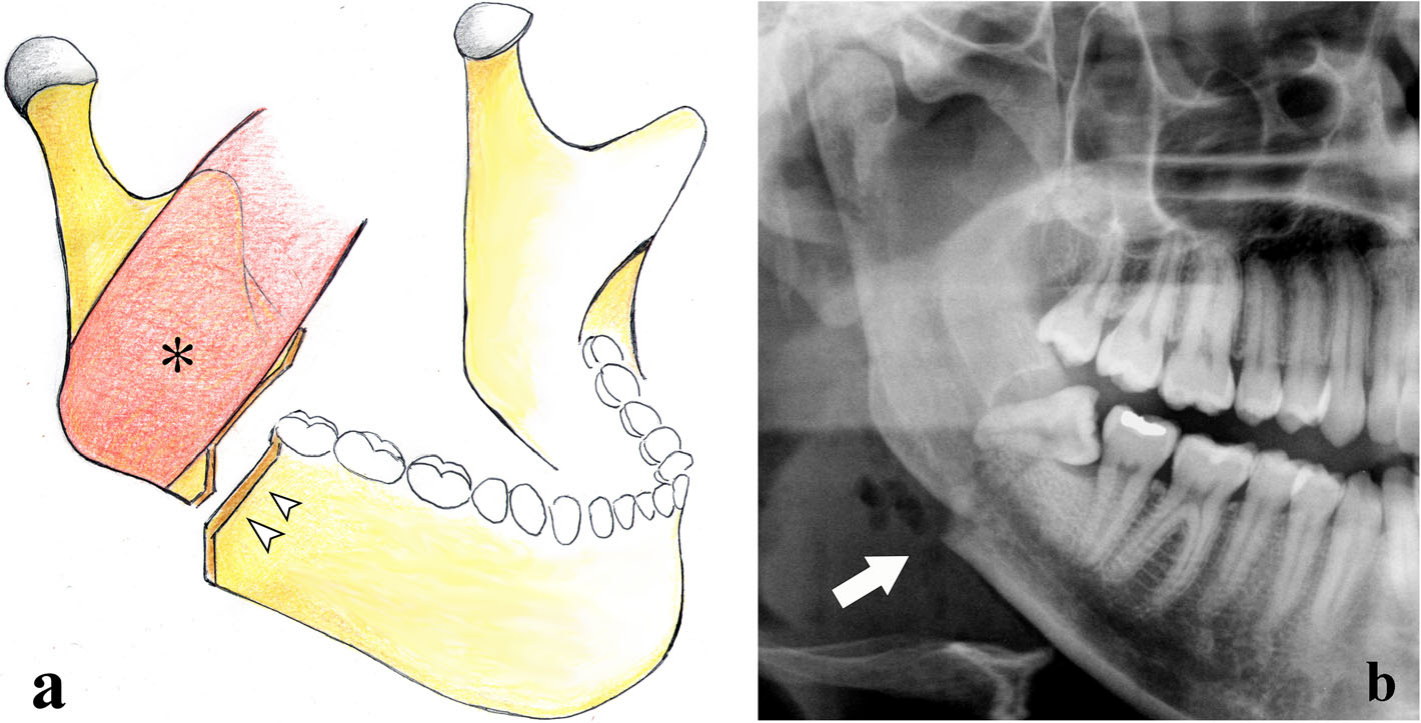
Angle fracture. **a** Picture showing a vertical fracture that runs distally to the third molar (arrowheads). It is a displaced fracture since the masseter muscle (asterisk) pulls the distal bone fragment upwards and medially. **b** Cropped panoramic radiograph. Mandibular angle fracture involving an impacted third molar (arrow)

Predisposing causes of mandibular angle fractures are represented by impacted wisdom teeth (Fig. 5) and conditions leading to a thinning/weakening of the mandible such as lytic lesions (cysts or tumours), osteoporosis, osteomyelitis, congenital hypoplasia, and toothless jaws.

### Mandibular ramus

The mandibular ramus corresponds to the anatomic area between the angle and the lower edge of the mandibular condyle. From the upper edge of the mandibular ramus arise two processes—the coronoid process anteriorly and the condylar process posteriorly—separated by a concavity named sigmoid notch. The external face of the mandibular ramus is flat; it is the masseter muscle attachment point. The medial pterygoid muscle is inserted in the lower internal portion of the mandibular ramus (Table 1).

**Fig. 6:**
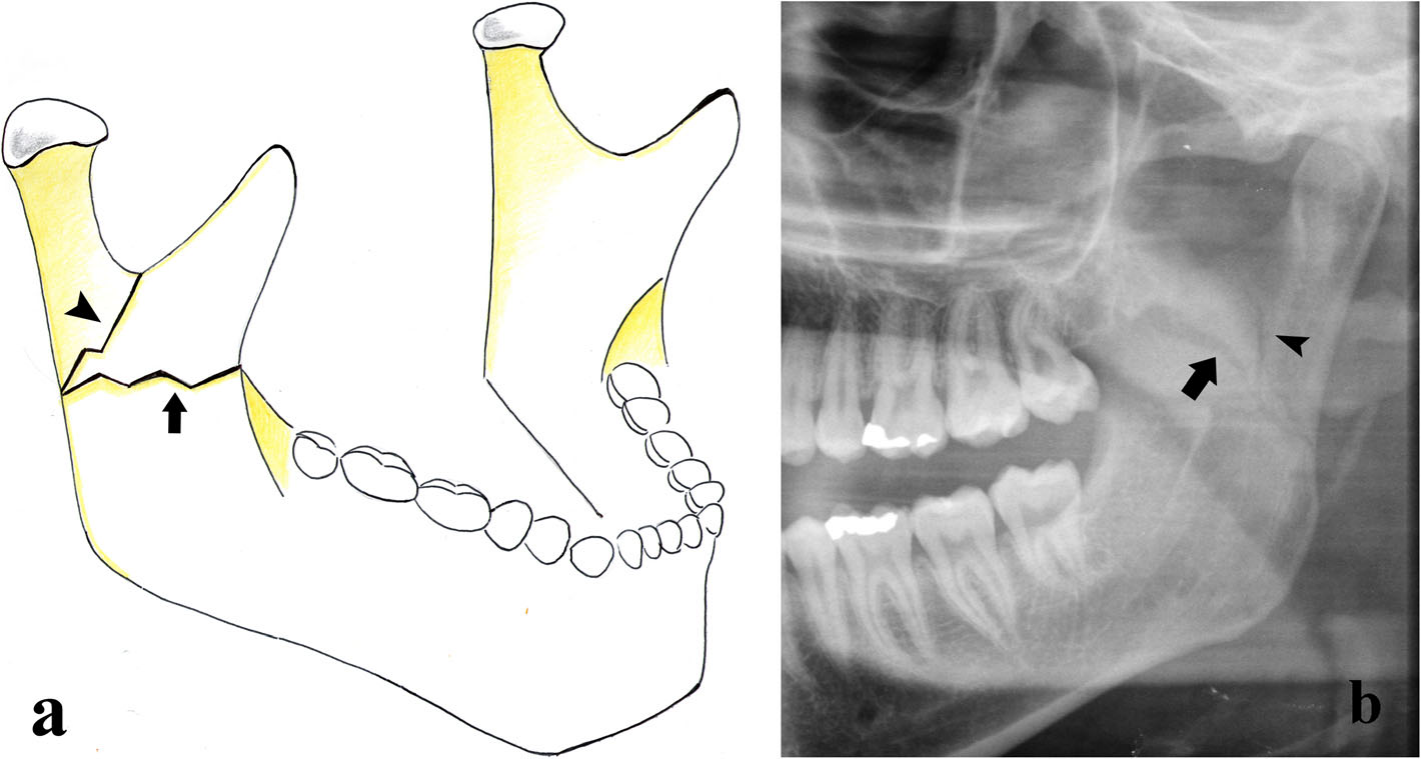
Ramus fractures. **a** Picture showing that the mandibular ramus fracture can be vertical (arrowhead) or horizontal (arrow), depending on the direction of the fracture rhyme. **b** Cropped panoramic radiograph. Combined fracture of the left mandibular ramus. The fracture rhyme originates from the external face of the ramus and has both a vertical (arrowhead) and a horizontal (arrow) course

Fractures of the mandibular ramus are commonly not solitary and are almost always due to direct and violent trauma. The fracture rhyme can have different directions, although it usually has a horizontal course. Few classifications of mandibular ramus fractures are found in the literature [5]. They are divided into vertical, horizontal, and combined fractures (Fig. 6).

**Fig. 7:**
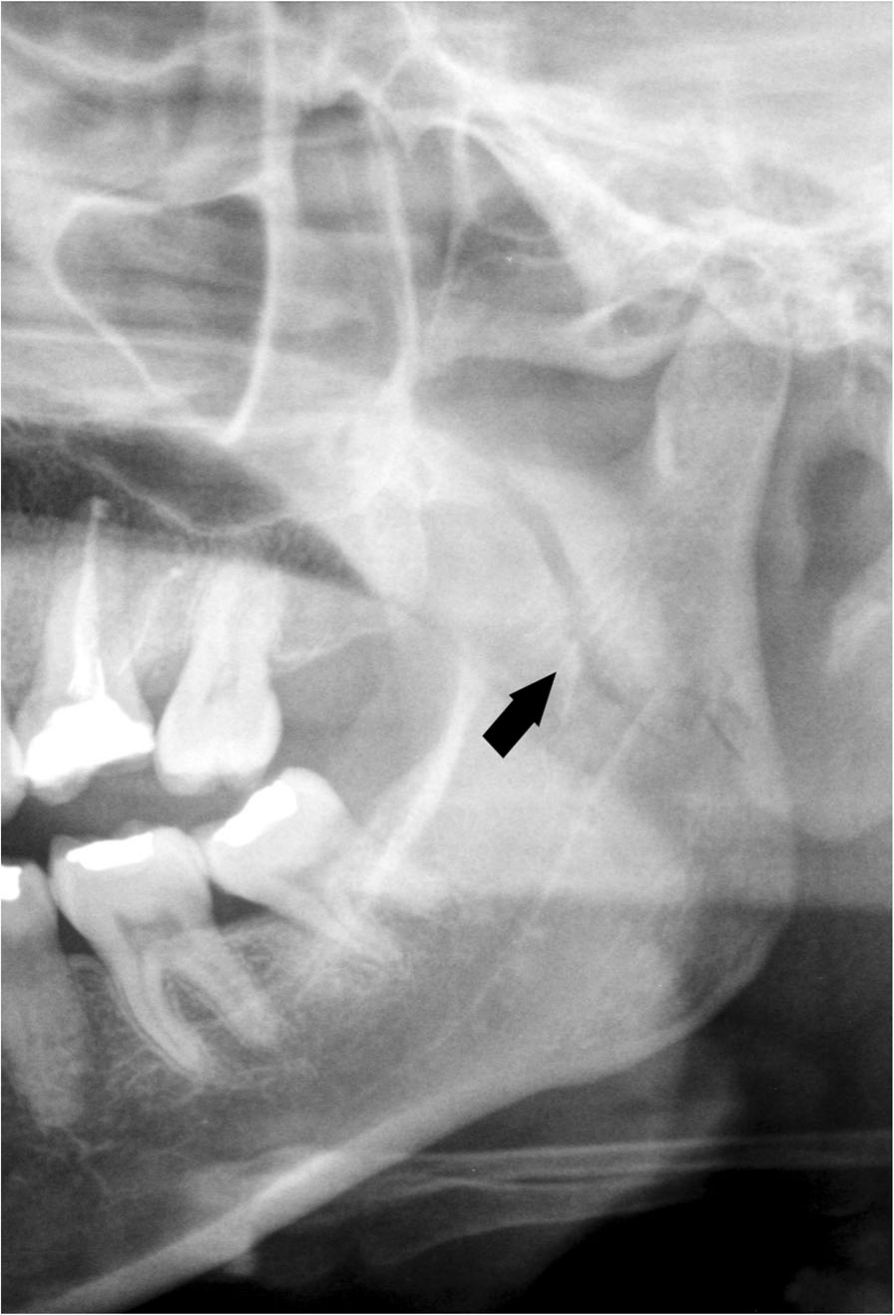
Vertical ramus fracture. Cropped panoramic radiograph showing a fracture of the left mandibular ramus, which runs from the external face of the ramus to the sigmoid notch (arrow)

- Vertical fracture. The fracture rhyme originates from the external face of the ramus and ends at the sigmoid notch (Fig. 7).
- Horizontal fracture. The fracture rhyme runs from the external face to the internal face of the ramus.
- Combined fracture. Both vertical and horizontal fractures are found.

### Coronoid process

The coronoid process is a thin triangular eminence located at the antero-superior end of the mandibular ramus. The coronoid process gives insertion to the temporal muscle and therefore contributes to the opening and closing of the mandible [24].

The coronoid process rarely faces fracture because it is well protected by several bone structures, especially the zygomatic complex. An isolated fracture of the coronoid process should be seen with suspicion and other concomitant mandibular fractures should be investigated [25].

**Fig. 8:**
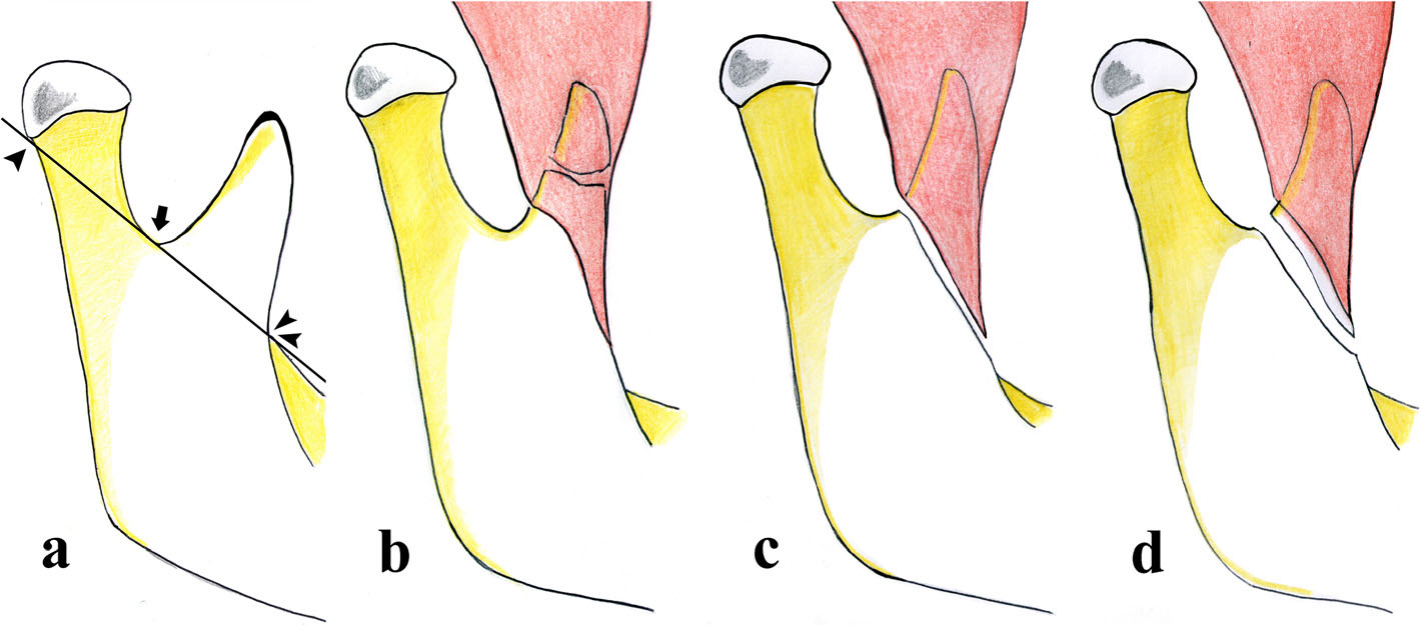
Coronoid process fracture. **a** A straight line passing through the deepest central point of the sigmoid notch (arrow) is traced from the lower posterior portion of the condylar head (single arrowhead) to the anterior edge of the mandibular ramus (double arrowhead). The bone portion included between the arrow and double arrowhead represents the coronoid process. **b** Coronoid process apex fracture. The fracture is fully included in the temporal muscle. Bone fragment displacement is minimal. **c** Coronoid process fracture medial to the deepest central point of the sigmoid notch. The fracture approximately originates in the correspondence of the temporal muscle attachment points. **d** Coronoid process fracture corresponding to the deepest central point of the sigmoid notch. The fracture originates below the temporal muscular attachment

**Fig. 9:**
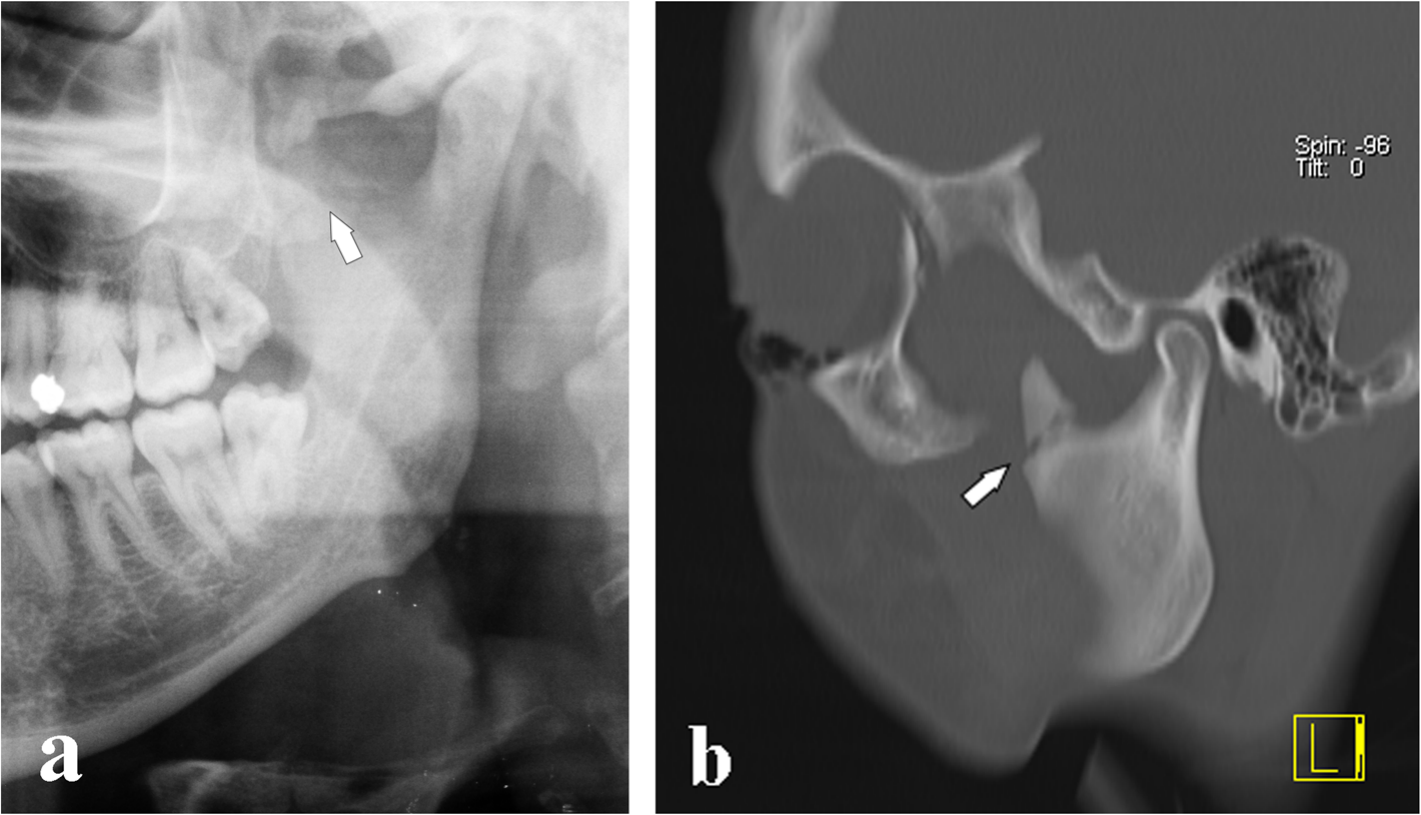
Coronoid process apex fracture. **a** Cropped panoramic radiograph. The fracture rhyme (arrow) runs from the internal to external faces of the coronoid process. **b** Computed tomography 5-mm oblique reconstruction of the same patient. Note that the fracture rhyme (arrow) ends higher than the sigmoid notch

**Fig. 10:**
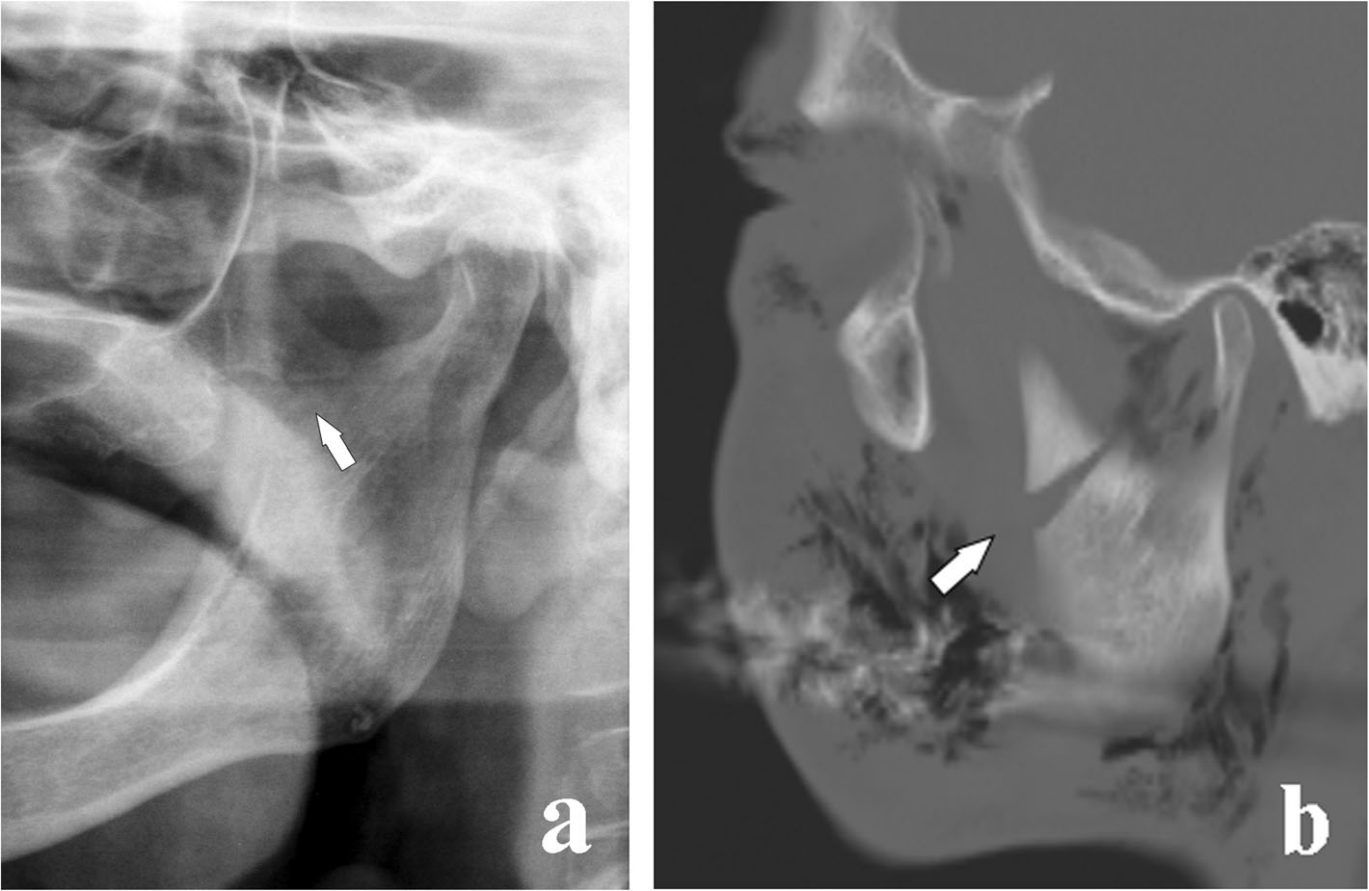
Coronoid process fracture medial to the deepest central point of the sigmoid notch. **a** Cropped panoramic radiograph in a toothless patient. The fracture rhyme (arrow) originates from the internal face of the coronoid process and ends medially to the deepest central point of the sigmoid notch. **b** Computed tomography 5-mm oblique reconstruction of a different patient who underwent the examination following a road accident. The displacement of the bone fragment is remarkable (arrow)

**Fig. 11:**
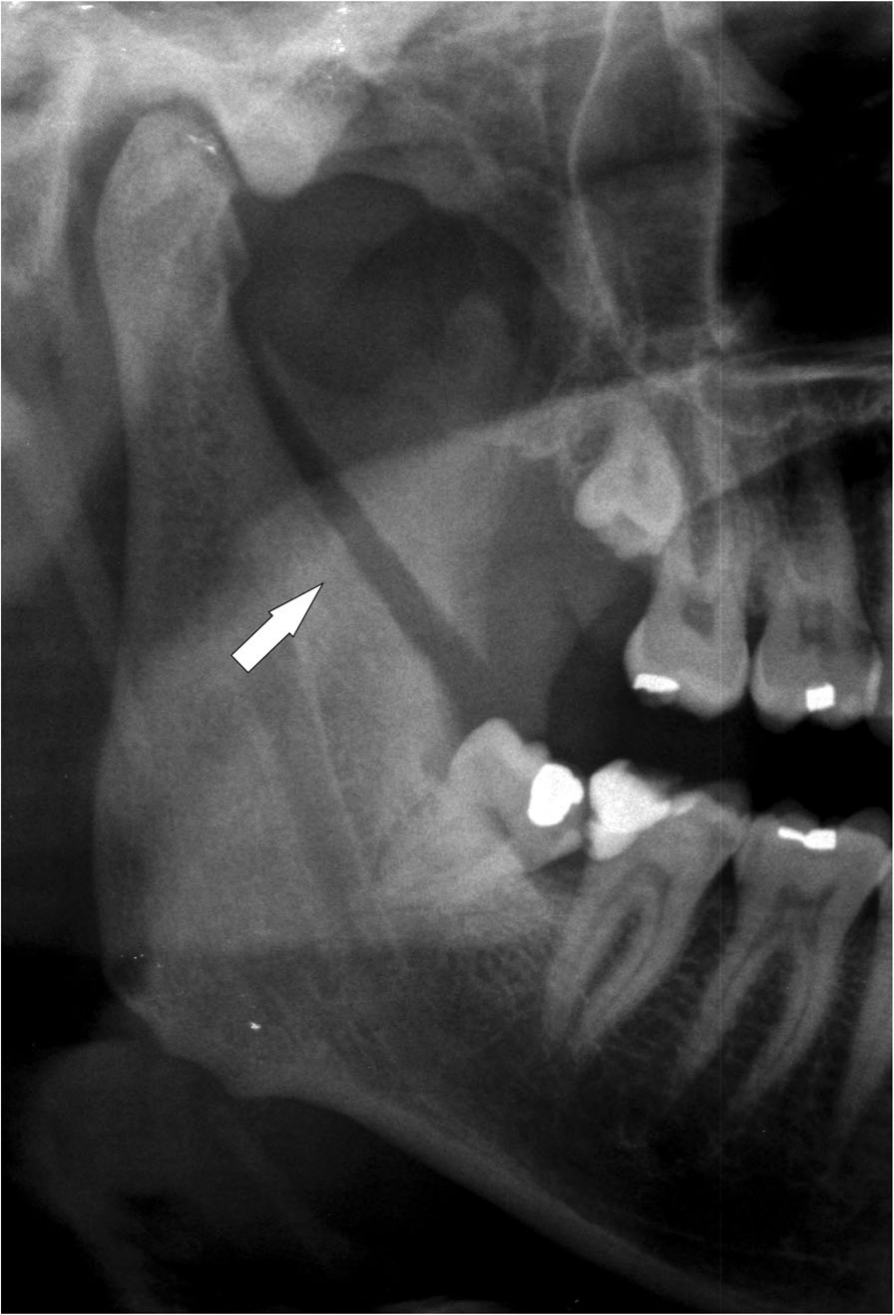
Coronoid process fracture lateral to the deepest central point of the sigmoid notch. Cropped panoramic radiograph showing a displaced fracture of the right coronoid process (arrow). The temporal muscle elevates the bone fragment upwards

Coronoid process fractures are due to direct trauma or a violent contraction of the temporal muscle. Based on the position of the fracture rhyme, the coronoid process fractures can be classified as follows [26] (Fig. 8):

- Coronoid process apex fracture (Fig. 9).
- Coronoid process fracture medial to the deepest central point of the sigmoid notch (Fig. 10)
- Coronoid process fracture corresponding or lateral to the deepest central point of the sigmoid notch (Fig. 11)

Coronoid process apex fracture is the most common coronoid process fracture. It is fully included in the temporal muscle tendon and the bone fragments are infrequently displaced, whereas the other two types of coronoid process fracture are submuscular fractures and therefore are more susceptible to induce a displacement of bone fragments [27].

### Condylar process

The condylar process consists of a head and a neck. The head of the condyle is articulated with the disc of the temporomandibular joint, while the neck is the narrow portion that supports the head. The anterior surface of the neck has a depression for the attachment of the lateral pterygoid muscle. The upper and lower heads of the lateral pterygoid muscle drag the disc forward and allow movements of lateral translation forward, respectively (Table 1). The temporomandibular joint is a condylarthrosis between the head of the mandibular condyle and glenoid fossa of the temporal bone.

**Fig. 12:**
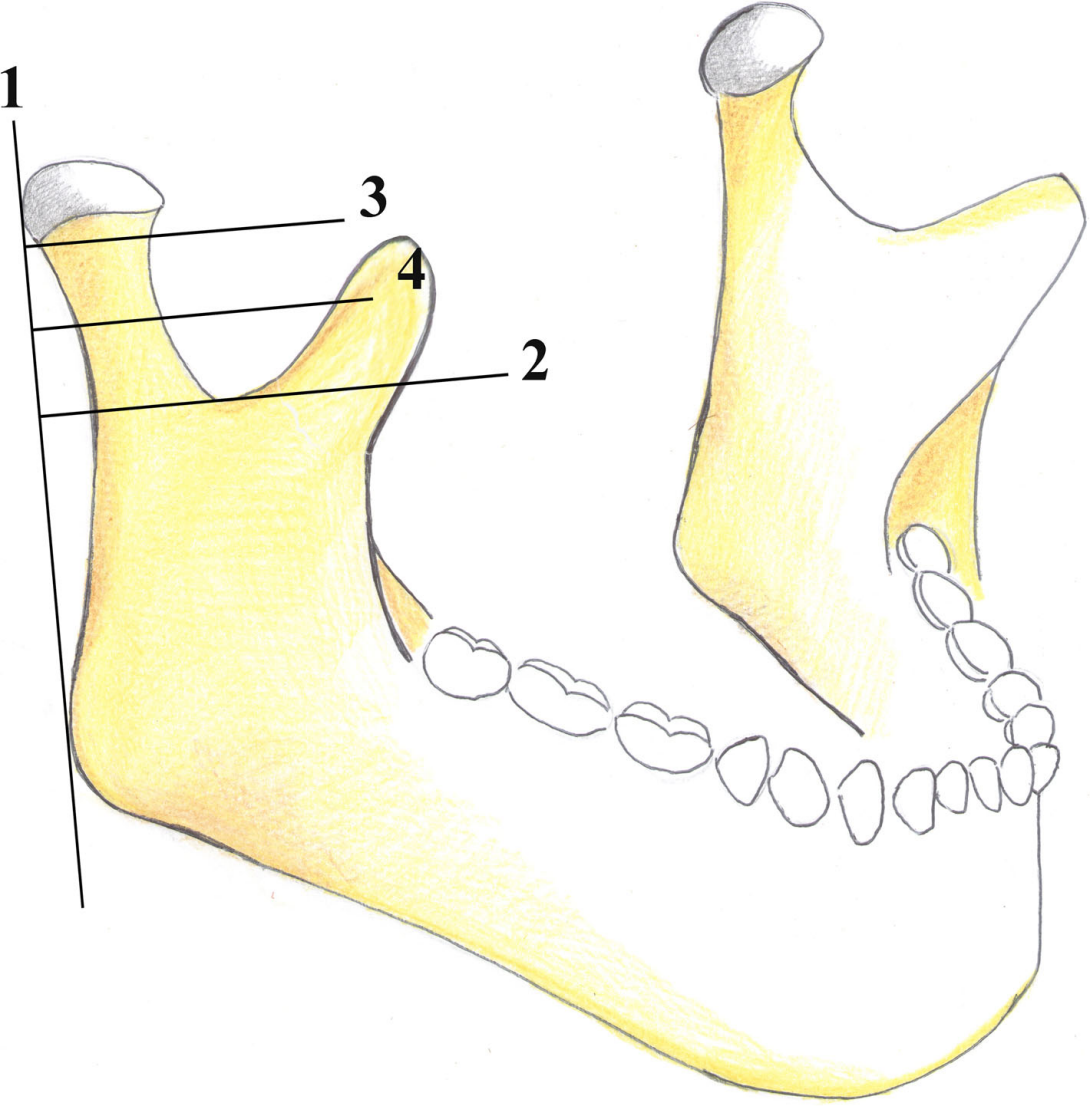
Picture depicting mandibular condyle fractures in according with the AO Foundation’s classification. A fracture is considered “high-neck” and “low-neck” when it is above and below Line 4, respectively. A full description is provided in the main text

There is no univocal consensus among authors on the classification of condylar fractures that should be used [28]. In our opinion, both the classifications given by the AO Foundation [29] and MacLennan et al. [30] should be used in a radiological report for an efficient and easily understandable subdivision of condylar fractures. The AO Foundation’s classification describes the fracture location. It divides the condylar fractures into three groups: head, “high-neck,” and “low-neck” fractures. The distinction between high- and low-neck can be achieved by drawing some lines on the image, as detailed below (Fig. 12):

- The first line runs tangent to the posterior edge of the condylar head and mandibular angle.
- The second line runs perpendicular to the first one passing through the sigmoid notch.
- The third line, perpendicular to the first one, passes through to the lower edge of the condylar head.
- The fourth line is in the middle between the second and third lines. A fracture is considered as a high- and low-neck fracture when it is above and below the fourth line, respectively.

**Fig. 13:**
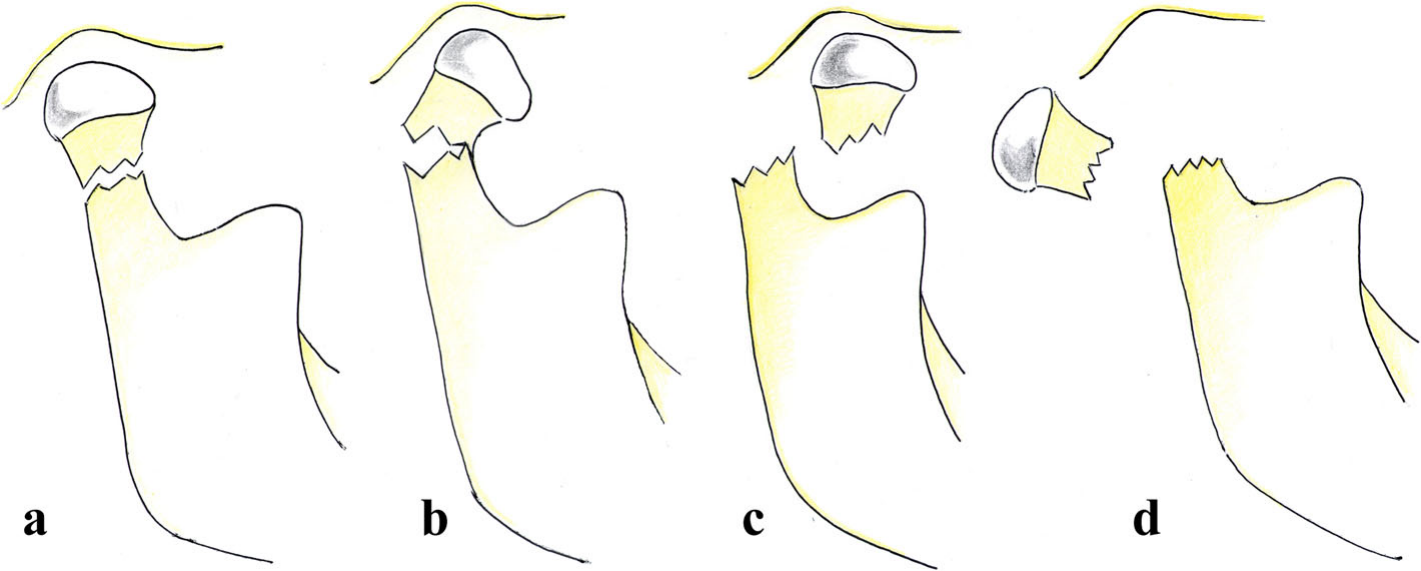
Picture depicting mandibular condyle fractures in according to the classification given by MacLennan et al. **a** No deviation. The bone fragments are in line and close to each other. The articular relation between the condylar head and glenoid fossa is maintained. **b** Deviation. A contact between the two bone fragments is observed but they are not in line. The articular relation between the condylar head and glenoid fossa is maintained. **c** Displacement. The condylar head remains within the glenoid fossa but there is no contact between the two bone fragments. **d** Dislocation. The articular relation between the condylar head and glenoid fossa is lost

**Fig. 14:**
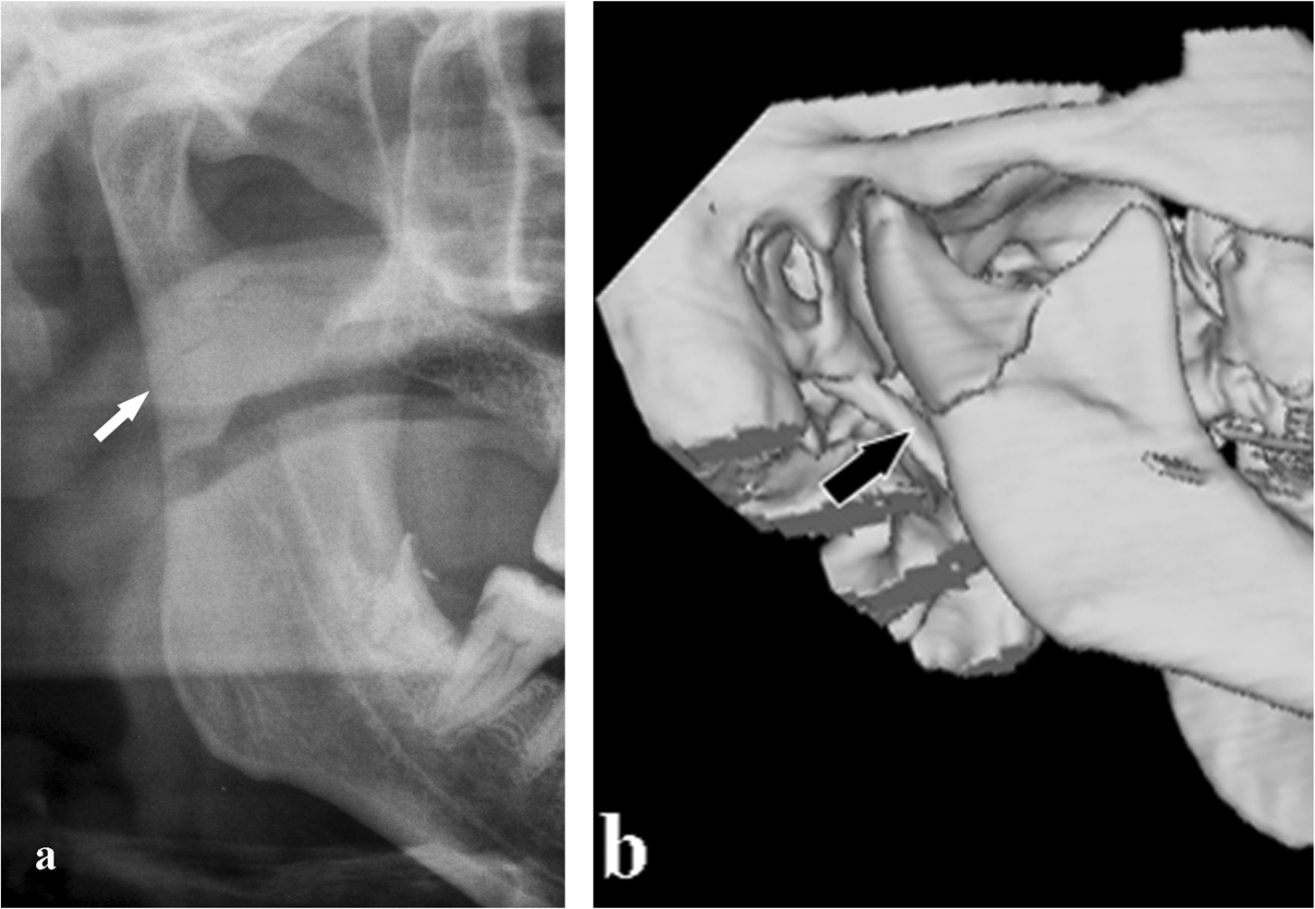
Condylar process fracture. No deviation. **a** Cropped panoramic radiograph. **b** 3D computed tomography reformation of the same patient. The bone fragments are in line and close to each other (arrows)

**Fig. 15:**
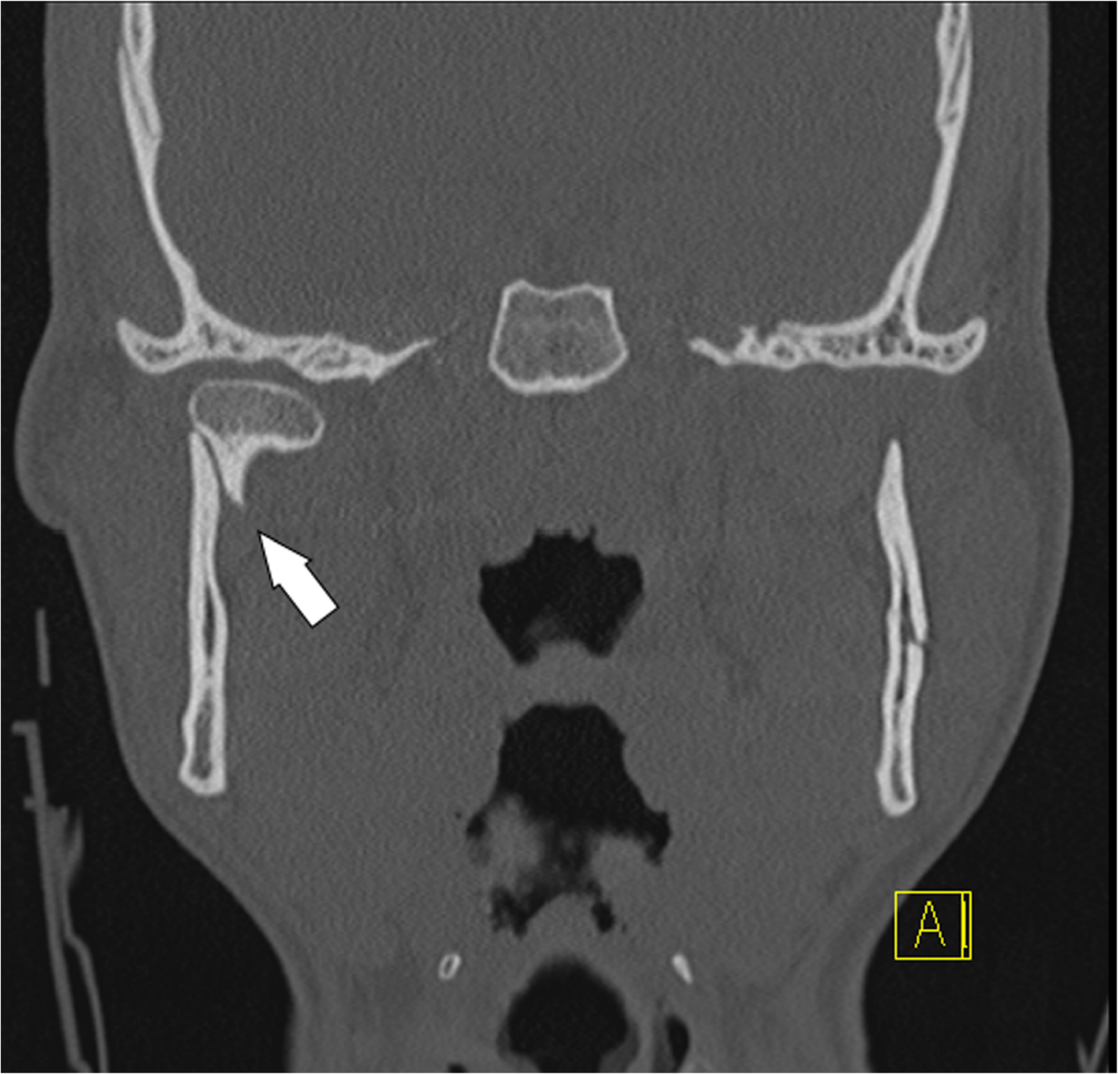
Condylar process fracture. Deviation. Computed tomography coronal section shows that the condylar head remains within the glenoid fossa and the contact between the bone fragments is not completely lost (arrow)

**Fig. 16:**
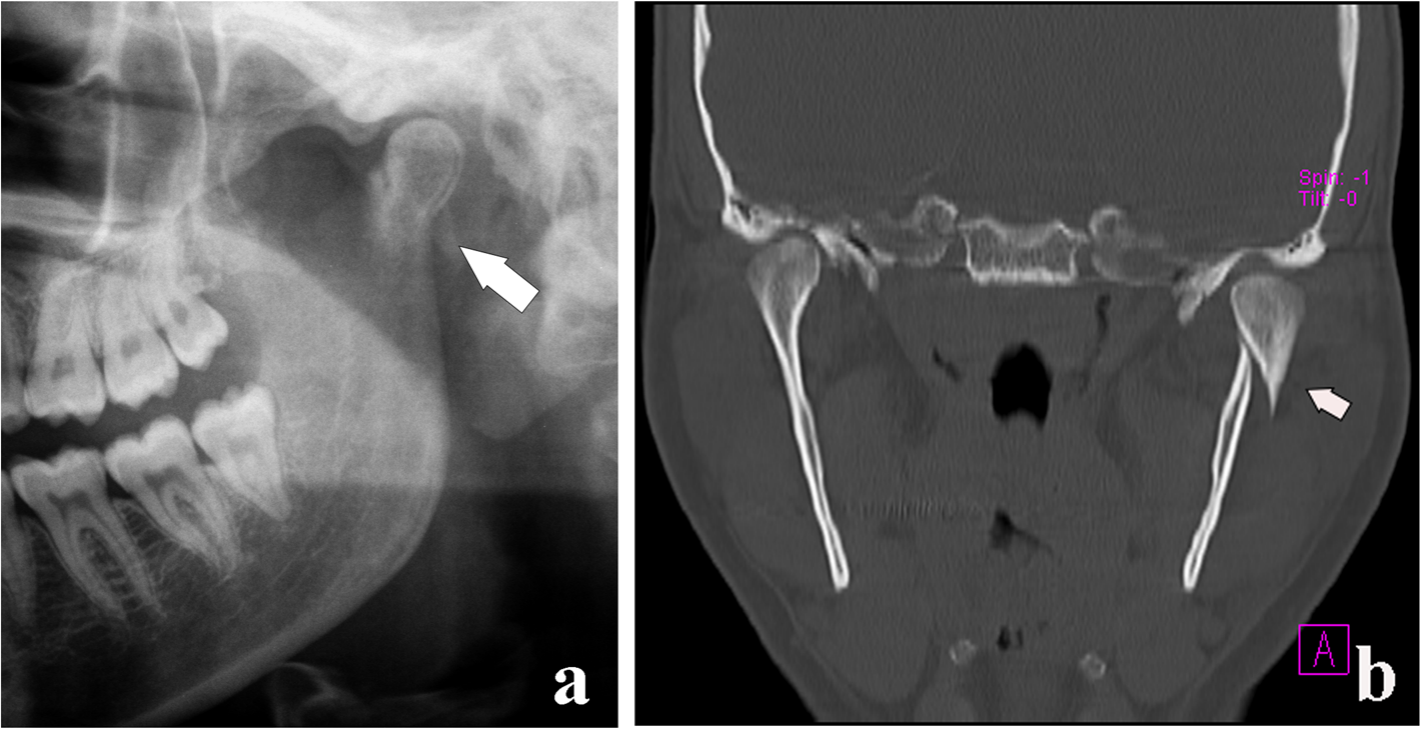
Condylar process fracture. Displacement. **a** Cropped panoramic radiograph. **b** Computed tomography coronal section of the same patient. The bone fragments are not in line (arrows), but the condylar head remains within the glenoid fossa

**Fig. 17:**
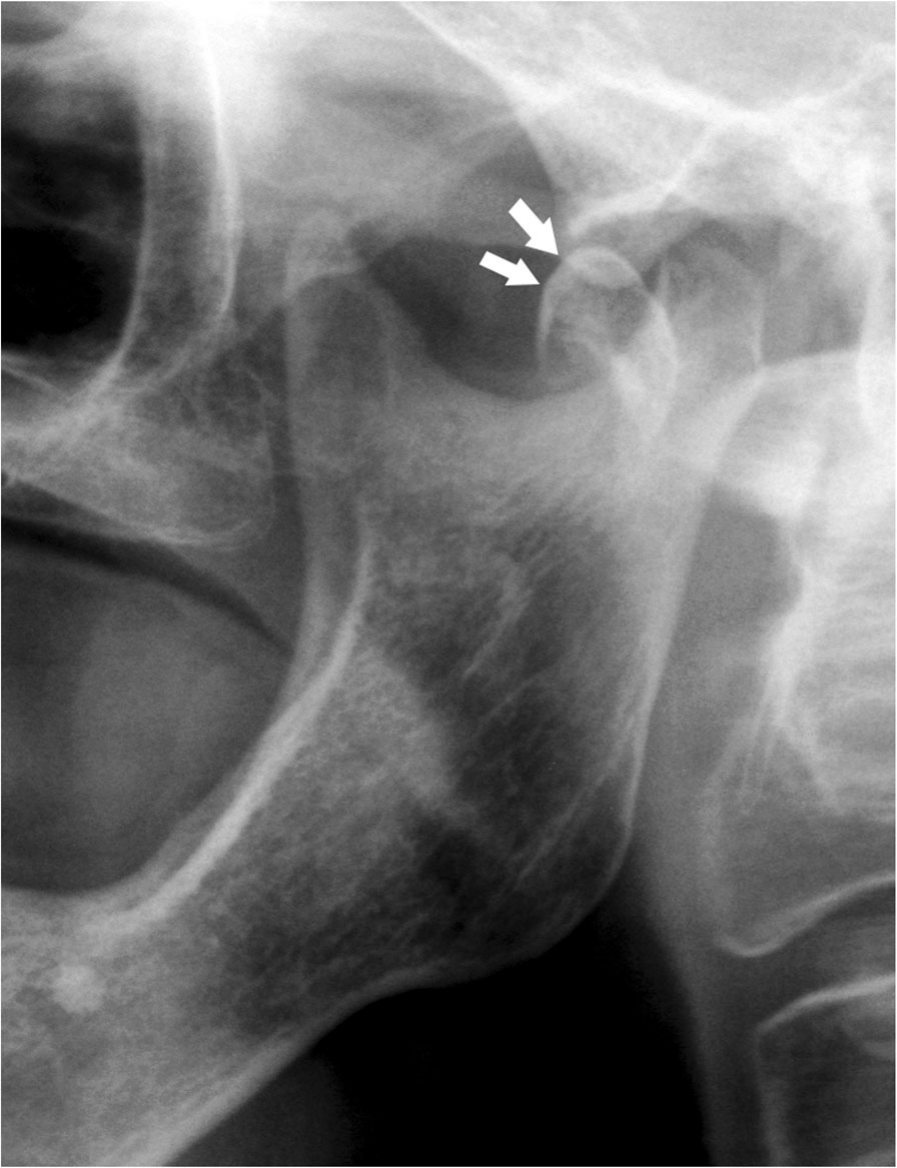
Condylar process fracture. Dislocation. Cropped panoramic radiograph. The bone fragments are not in line and the condylar head moves out from the glenoid fossa (arrows)

**Fig. 18:**
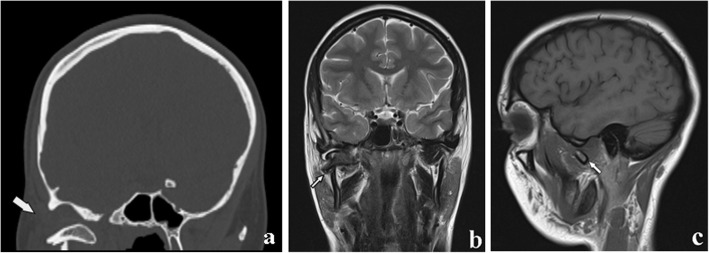
Condylar process fracture. Dislocation. **a** Computed tomography coronal section. **b**, **c** Magnetic resonance T2w coronal image and T1w sagittal image of the same patient. The condylar head is in the horizontal position and completely outside the glenoid fossa (arrows)

MacLennan et al.’s classification describes the displacement of bone fragments (Fig. 13) as follows:

- No deviation (no bending) (Fig. 14)
- Deviation (bending). A fracture where contact between the two bone fragments is preserved (Fig. 15).
- Displacement. The condylar head remains within the glenoid fossa; nevertheless, a loss of contact between the bone fragments is found (Fig. 16).
- Dislocation. The condylar head comes out of the glenoid fossa (Figs. 17 and 18).

**Fig. 19:**
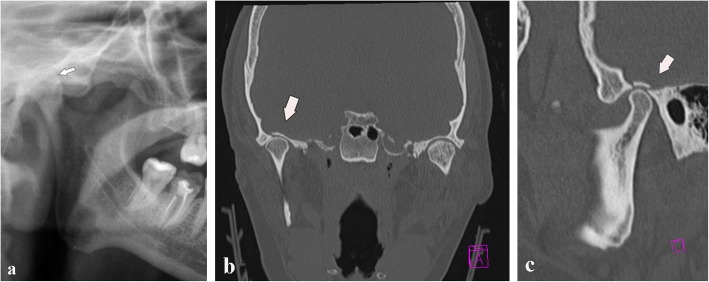
Fracture of the glenoid fossa of the temporal bone. **a** Cropped panoramic radiograph shows a reduction of the articular space. The condylar head seems to be higher than its usual location (arrow). **b**, **c** Computed tomography coronal and sagittal sections. A slight upward movement of the mandibular condyle associated with a glenoid fossa fracture of the temporal bone is observed (arrows)

The condylar neck is the weakest area of the mandible. It responds to the need to defend the middle cranial fossa from the traumatic energy that would be transmitted to it by the mandibular condyle. The interruption of the traumatic energy at the site of the condylar neck is a means of defence for the endocranium. In fact, only few cases of glenoid fossa fractures and endocranial dislocation of mandibular condyles have been described [28] (Fig. 19). Moreover, the fracture of both condylar necks is common when the trauma is applied to the chin symphysis.

**Fig. 20:**
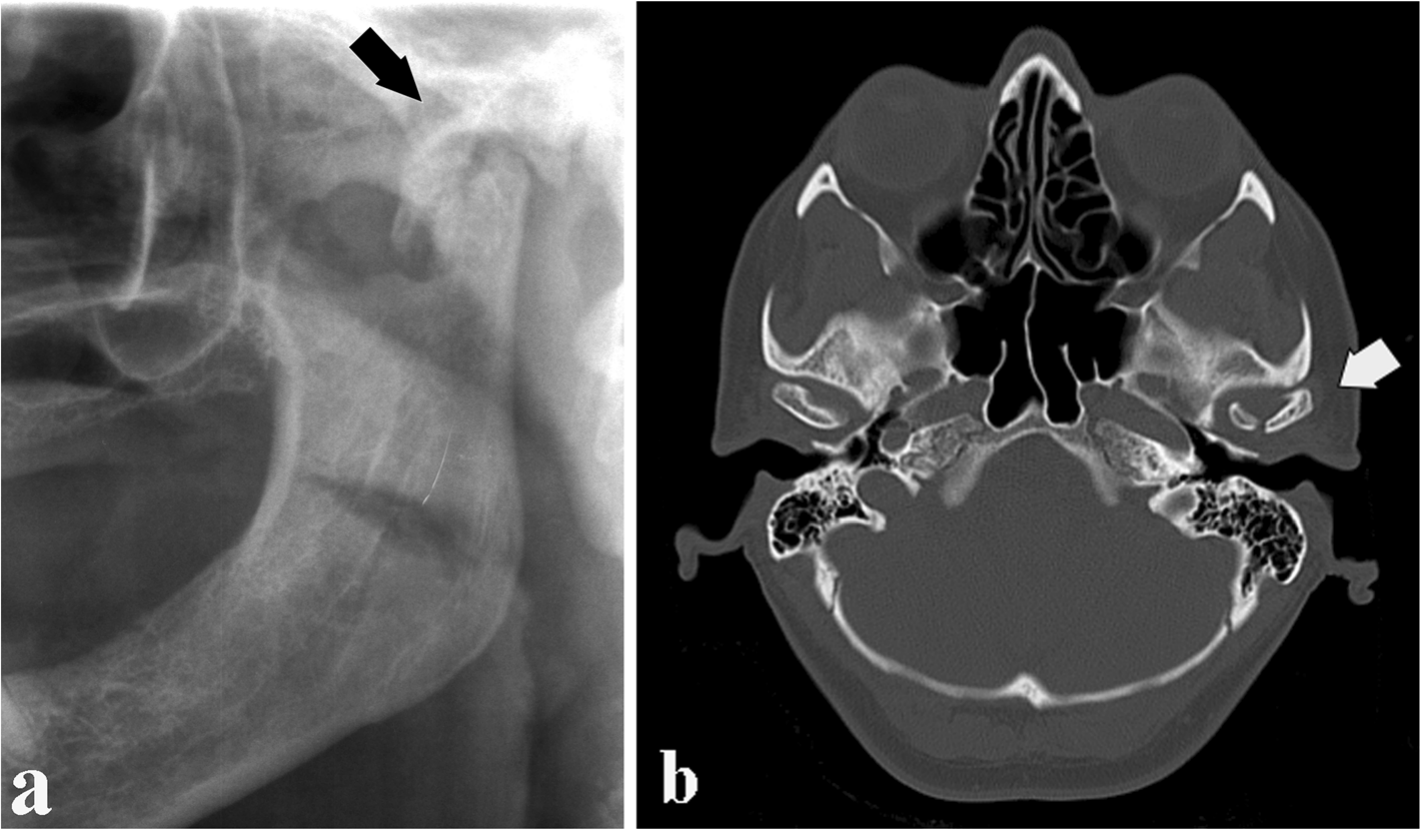
Condylar head fracture. **a** Cropped panoramic radiograph. **b** Computed tomography axial section. Oblique fracture of the condylar head with involvement of the articular surface (intracapsular fracture) (arrows)

Condylar head fractures are rarer than condylar neck fractures. Condylar head fractures are due to a direct trauma from the bottom to the top on the mandibular angle, which causes crushing of the condyle on the temporal bone (Fig. 20).

## Mandibular fractures treatment

Surgical treatments are aimed to restore the anatomy and function of the mandible by immobilising and realigning the fractured bones. Therapeutic approaches range from non-invasive conservative management by “closed” reduction and immobilisation with intermaxillary fixations to “open” surgical reduction with internal fixations [31].

Numerous factors influence the treatment of mandibular fractures, including the location and degree of fragment displacements, patient’s age/health, and surgeon’s ability. In the “closed” (non-surgical) reduction, the bone fragments are realigned manually or by using traction devices without surgically exposing the fracture site. The open reduction surgery of mandibular fractures should first ensure the restoration of the occlusion of the mandible to prevent postoperative malocclusion, followed by stabilisation by means of rigid fixations such as plates, screws, and rigid intermaxillary blocks in order to minimise any nonunion, malunion, or delayed union of the fracture segments.

**Fig. 21:**
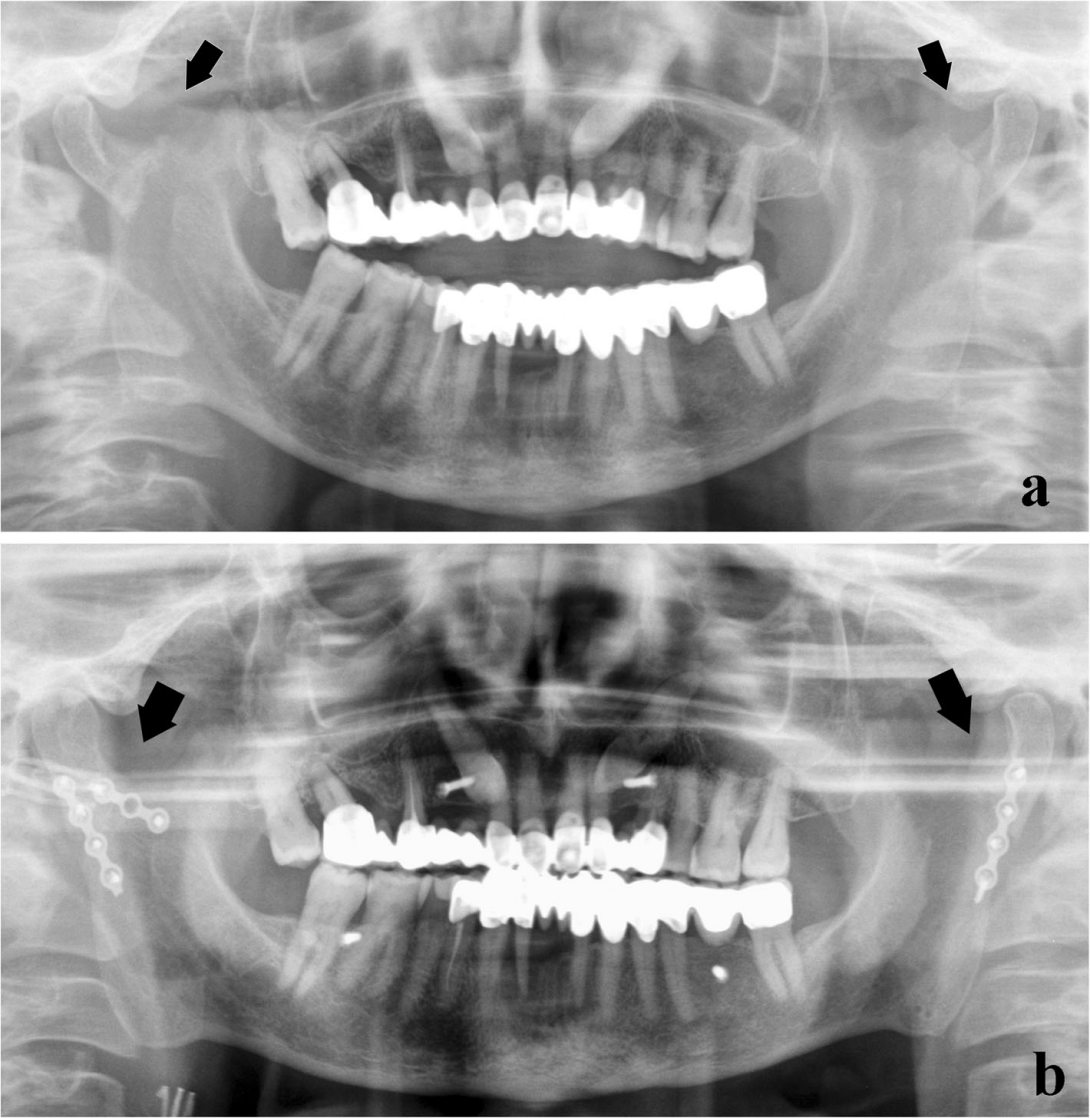
Bilateral condylar process fracture with extracapsular lateral dislocation. **a** Panoramic radiograph shows a fracture of both condylar necks (arrows), following a violent trauma on the chin symphysis. **b** The lateral displacement of the condylar heads was subjected to “open” reduction and rigid internal fixations with osteosynthesis plates (arrows)

General indications for a “closed” reduction in mandibular fractures are as follows [1, 32, 33]:

- Paediatric fractures. An “open” reduction may damage developing dental gems or partially erupted teeth.
- Coronoid process fractures. They are rarely treated, unless an impingement on the zygomatic arch is found.
- Condylar process fractures. Their treatment is a controversial issue. The most appropriate choice is generally a conservative treatment, unless certain specific conditions mandatorily need an “open” treatment [34], such as condylar shifts into the middle cranial fossa or external auditory canal, failures in appropriate occlusion, extracapsular lateral dislocations of the condyle (Fig. 21), and infected open joint lesions.

If a fracture of the mandibular condyle does not meet the criteria above, patients can be treated with “closed reduction” for 2–3 weeks.

**Fig. 22:**
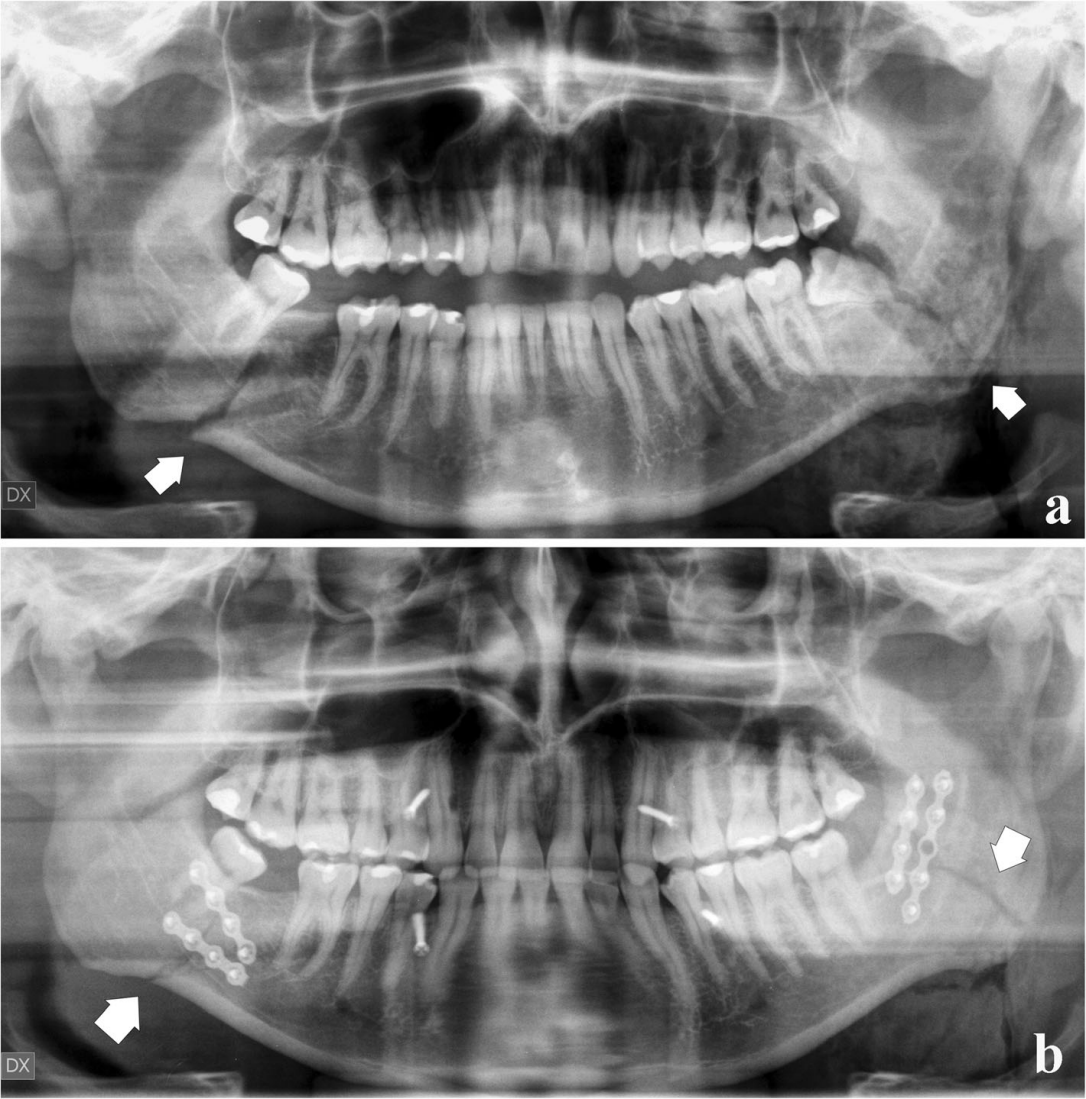
Bilateral angle fracture. **a** Panoramic radiograph shows fractures of both mandibular angles (arrows) involving the lower third molars. **b** After removing the lower left third molar, rigid internal fixations with osteosynthesis plates merge the bone segments

The mandibular fractures that usually require an “open” reduction are as follows [23]:

- Mandibular angle fractures, especially if bone fragments are misaligned (Fig. 22)
- Atrophic toothless mandible, poor osteogenesis, or reduced healing potential
- Complex maxillofacial fractures

Assessment of mandibular fractures by imaging techniques is crucial for directing the patient towards surgical or conservative treatments.

## Summary

The aim of imaging techniques is to identify the presence, number, and exact localisation and extension of fracture rhymes, as well as to analyse the concomitant complications in the adjacent anatomical structures. The therapy may be a conservative or surgical treatment based on the site and fracture characteristics.

## Data Availability

All data generated and analysed during the current study are included in this published article. Moreover, the datasets are available from the corresponding author on reasonable request.

## Abbreviations

CBCT: Cone beam computed tomography
MRI: Magnetic resonance imaging
MSCT: Multislice spiral computed tomography
PAN: Panoramic radiography
